# Molecularly Imprinted Polymers for the Removal of Antide-Pressants from Contaminated Wastewater

**DOI:** 10.3390/polym13010120

**Published:** 2020-12-30

**Authors:** Tjasa Gornik, Sudhirkumar Shinde, Lea Lamovsek, Maja Koblar, Ester Heath, Börje Sellergren, Tina Kosjek

**Affiliations:** 1 Department of Environmental Sciences, Jozef Stefan Institute, Jamova 39, 1000 Ljubljana, Slovenia; tjasa.gornik@ijs.si (T.G.); ester.heath@ijs.si (E.H.); 2 Jozef Stefan International Postgraduate School, Jamova 39, 1000 Ljubljana, Slovenia; maja.koblar@ijs.si; 3 Department of Biomedical Sciences and Biofilms-Research Center for Biointerfaces (BRCB), Faculty of Health and Society, Malmö University, 20506 Malmö, Sweden; sudhirshinde1@gmail.com (S.S.); borje.sellergren@mau.se (B.S.); 4 School of Chemistry and Chemical Engineering, Queens University Belfast, Belfast BT9 5AG, UK; 5 Department of Biopharmacy and Pharmacokinetics, Faculty of Pharmacy, University of Ljubljana, Askerceva 7, 1000 Ljubljana, Slovenia; lealamovsek@gmail.com; 6 Center for Electron Microscopy and Microanalysis (CEMM), Jamova 39, 1000 Ljubljana, Slovenia

**Keywords:** molecular imprinting, polymer, wastewater treatment, sertraline, cross-reactivity, SSRI, template, sorbent

## Abstract

Selective serotonin reuptake inhibitors (SSRIs) are a class of antidepressants regularly detected in the environment. This indicates that the existing wastewater treatment techniques are not successfully removing them beforehand. This study investigated the potential of molecularly imprinted polymers (MIPs) to serve as sorbents for removal of SSRIs in water treatment. Sertraline was chosen as the template for imprinting. We optimized the composition of MIPs in order to obtain materials with highest capacity, affinity, and selectivity for sertraline. We report the maximum capacity of MIP for sertraline in water at 72.6 mg g^−1^, and the maximum imprinting factor at 3.7. The MIPs were cross-reactive towards other SSRIs and the metabolite norsertraline. They showed a stable performance in wastewater-relevant pH range between 6 and 8, and were reusable after a short washing cycle. Despite having a smaller surface area between 27.4 and 193.8 m^2^·g^−1^, as compared to that of the activated carbon at 1400 m^2^·g^−1^, their sorption capabilities in wastewaters were generally superior. The MIPs with higher surface area and pore volume that formed more non-specific interactions with the targets considerably contributed to the overall removal efficiency, which made them better suited for use in wastewater treatment.

## 1. Introduction

The fast population growth, advances in industry, and increased agricultural activity have greatly influenced the environment. In order to continue with the current pace, we need solutions in environmental management, especially wastewater (WW) reuse. The development in the area of sample preparation and instrumentation has put the removal of trace-level emerging contaminants in the forefront of environmental research [1,2]. Among them, pharmaceuticals are a very problematic group, since they are particularly designed to have a pharmacological effect on humans or animals, thus potentially yielding adverse effects in living organisms [3] after they have entered the aquatic environment. 

The selective serotonin reuptake inhibitors (SSRIs) are members of the most prescribed class of antidepressants in the USA and Europe [4,5,6]. They have been repeatedly detected in WW, surface waters, sediments, and aquatic organisms [7,8,9,10,11,12], and are thus part of different monitoring programs [13]. In aquatic organisms, SSRIs cause changes in biochemical processes, feeding behavior, survivorship behavior, growth, and potential changes in their genetic material [7,14,15,16,17]. Hence, it is crucial to improve their removal from WW before they are introduced into the environment. Among the existing WW treatment techniques, advanced oxidation processes and biological treatment are most successful in removing SSRIs from WW [18,19]. While during the former, the leading process of removal is degradation, sorption to activated sludge seems to be responsible for the removal of the majority of SSRIs during biological treatment [19]. Hence, other adsorption-based treatment techniques have been considered. Among them, activated carbon (AC) is by far the most researched material for SSRI removal [20,21]. AC as a treatment technique is technologically simple, has relatively fast kinetics, and removes a high variety of contaminants. Its main disadvantages are a high initial investment, the non-selectivity of the process, and the need for frequent regeneration due to fouling, which is expensive, time-consuming, and results in the loss of material in each regeneration cycle [22,23]. Greener alternatives, such as using products of pyrolysis of primary and secondary paper mill sludge, spent coffee grounds, and pine bark have been reported [24,25]. However, there is a lack of literature investigating modified synthetic composite materials, such as carbon-based nanomaterials, different types of membranes, and other forms of modified polymers, which, however, present promising alternatives to achieve superior SSRI removal from WWs [19,26,27,28,29]. On the basis of this knowledge gap, we investigated molecularly imprinted polymers (MIPs) as an alternative sorption material to AC [23,30,31].

MIPs are polymers that have been imprinted by a chosen template during the polymerization step in order to create selective recognition sites and are therefore often referred to as artificial antibodies or synthetic receptors [32]. After the template is removed from the MIP, the same or similar molecule can be rebound. They have already been commercially used for solid-phase extraction (SPE) [33,34] and researched for several other applications, such as catalysis, chromatography, and drug delivery [35,36]. In the last few years, the number of studies considering MIPs for water treatment has increased. Thus far, they have been utilized to remove non-steroidal anti-inflammatory drugs, antibiotics, antimicrobials, endocrine-disrupting compounds, herbicides, phenols, and beta-blockers from contaminated WW [30,35,37,38,39,40,41]. The advantages of using MIPs for water treatment are their high selectivity and affinity for their targets. Hence, we expect to be able to regenerate the material after longer intervals compared to AC, since slower fouling rates are expected. Literature reports MIPs as mechanically and chemically stable, and thus they should withstand several regeneration cycles unchanged, making the treatment more cost-effective [35,38]. The main disadvantage of MIPs is, however, the initial investment into the production of the polymers. Among multiple polymerization procedures available today, we chose bulk polymerization as one of the simplest and cheapest one for MIP production [2].

**Figure 1:**
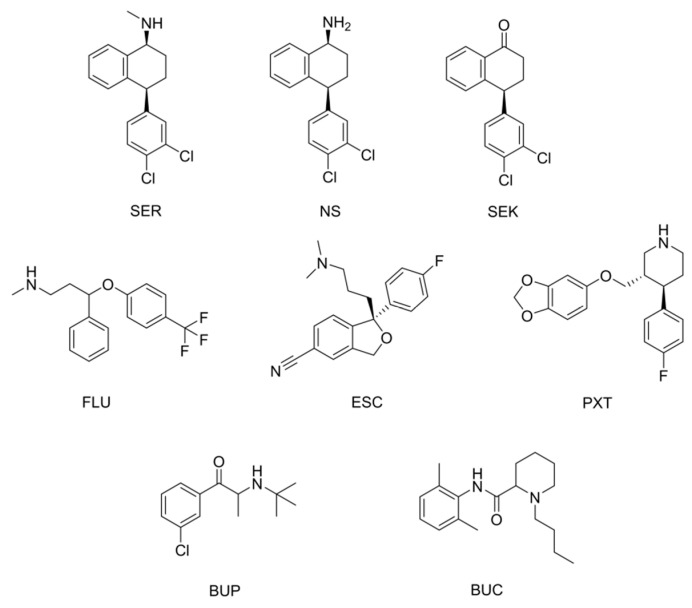
Chemical structures of the tested compounds.

The aim of this work was to develop a MIP that could be used for removal of not only our targeted template, but for the whole class of SSRIs. We evaluated the affinity, capacity, and selectivity of the synthetized MIPs for sertraline (SER) and chose the best performing materials. Further characterization included cross-reactivity towards other antidepressants fluoxetine (FLU), paroxetine (PXT), escitalopram (ESC), bupropion (BUP), two SER metabolites—norsertraline (NS) and sertraline ketone (SEK) [9,10,42], and structurally related compound bupivacaine (BUC) (Figure 1). Potential parameters influencing the removal were considered and the performance of the MIPs in WW was tested in order to evaluate their applicability for WW treatment. The composition of the polymers was confirmed using Fourier transform infrared spectroscopy (FTIR) and elemental analysis. Surface properties and pore volume were calculated on the basis of the obtained Brunauer–Emmett–Teller (BET) isotherms, and scanning electron microscopy images of materials were taken for morphological characterization.

## 2. Materials and Methods

The list of chemicals, materials, and the description of standard solution preparation and pre-preparation of the polymerization ingredients are reported in the Supplementary Material (SM) Section 1.

### 2.1. The Synthesis of MIP 

The polymers were prepared via bulk radical polymerization with the ingredients in ratios specified in Table 1.

The mini-MIP library was synthesized by varying functional monomer, porogen, and the form of the template, as illustrated in the Table 1. The molar ratio between the template, functional monomer, and cross-linker was 1/4/20. In the case of mini-MIPs, 34.1 mg (0.1 mmol) of sertraline in HCl salt form (SER HCl) or 30.8 mg (0.1 mmol) free base sertraline (SER), 34 µL (0.4 mmol) methacrylic acid (MAA), and 380 µL (2 mmol) ethylene glycol dimethacrylate (EGDMA) was used. A total of 560 µL of porogen (either CHCl_3_, methanol—MeOH, or acetonitrile—ACN) was added, with the exception of anhydrous toluene, where 580 µL was needed due to solubility issues. For polymers prepared using two functional monomers, we changed the ratio to 1/4/8/12 for the template (30.8 mg SER, 0.1 mmol), functional monomer (34 µL MAA, 0.4 mmol), co-monomer (860 µL of methyl methacrylate (mMA) or 970 µL of 2-hydroxyethyl methacrylate (HEMA), 0.8 mmol), and 227 µL of the EGDMA cross-linker (1.2 mmol). We used 1 wt % of the initiator 2,2’-azobis(2,4-dimethyl valeronitrile) (V-65) for synthesis of polymers on the basis of total monomers.

The synthetic procedure was identical for all MIPs. The monomers and the template were first mixed and dissolved in the porogen solvent. Then cross-linker EGDMA was added and the solution was mixed again. Finally, the initiator V-65 was added. The solution was mixed, purged with N_2_ for 10 min, and polymerized at 50 °C for 24 h in an oven. After 24 h, the polymerization was carried out for another 2 hours at 70 °C. The corresponding non-imprinted polymers (NIPs) were prepared following the identical procedures in the absence of the template.

Best-performing MIPs and their corresponding NIPs were later prepared in a 10 times larger quantity, maintaining the same polymer compositions and ingredients. The polymers were then crushed and sieved into 25–50 µm particle size. Both MIPs and NIPs underwent Soxhlet extraction in 10% of acetic acid in methanol for 96 h until no SER was detected by a high-performance liquid chromatograph coupled with a diode array detector (HPLC-DAD). The polymers were further washed with water and MeOH to remove the acetic acid, before drying them in the oven at 50 °C for 24 h. The dried polymers were used for further physical and analytical characterization.

### 2.2. Selection of the Material: Batch Rebinding

Batch rebinding tests were performed in both water and acetonitrile (ACN). A total of 5 mg of each MIP and the corresponding NIP was weighed and placed in 1.5 mL Eppendorf tubes containing 500 µL of the SER solution with increasing concentrations: 0.1, 0.4, 1.0, 2.0, 3.0, and 4.0 mM. We used SER × HCl for rebinding in water, and SER in the free base form for the rebinding in ACN. All the experiments were performed after the equilibrium had been reached, i.e., after 20 h (see Section 2.5). The suspension was centrifuged at 10,000 rpm for 15 min. The supernatant was diluted 10 times with the mixture of 50% ACN and 50% 20 mM phosphate buffer at pH 3.70 (mobile phase) and subsequently quantified by HPLC-DAD analysis. The levels of bound compounds to the MIP/NIP for each solvent mixture were estimated from plotted calibration curves. We plotted the data in the form of rebinding isotherms using the bi-Langmuir isotherm as the best fit (*R*^2^ > 0.90). The capacity, affinity, and selectivity were calculated for each polymer. Capacity was reported as the mass of bound compound per gram of polymer. Affinity was determined as the distribution ratio (D), the ratio between the amount of SER bound to the polymer (B), and the remaining SER in the supernatant (F). The selectivity was calculated as the imprinting factor (IF), comparing the D of MIP to the D of its corresponding NIP. All the parameters were calculated at equilibrium at the highest added concentration of 4.0 mM. On the basis of the results in both ACN and water, we chose three best performing MIPs for further testing.

### 2.3. Reusability Experiments

Reusability of the chosen MIPs and NIPs was tested by repeating 4 times the batch rebinding of 0.1 mM SER in ultrapure water (UW) on the same material, while following any changes in the performance. Between the cycles, the polymers were washed with 1 mL 1% trifluoroacetic acid (TFA) in MeOH (30 min) and 1 mL of MeOH (15 min) in order to remove SER from polymers. Solvent-free polymers were obtained by drying in the oven for 1 h at 60 °C. The experiment was performed in 5 parallels.

### 2.4. Cross-Reactivity Experiments

The cross-reactivity of the 3 materials selected as described in Section 2.2 was evaluated by binding experiments for antidepressants and their structurally related compounds: NS, SEK, FLU, ESC, PXT, BUP, and BUC. The cross-reactivity was assessed through selectivity factor (α), the capacity, and the difference in binding between MIP and NIP for each compound. A was calculated as the ratio between the D of SER and D of the tested compound.

The cross-reactivity experiments were performed separately for each compound in UW, applying the same conditions as for SER rebinding tests (see Section 2.2.). The experiments were performed at the concentration of 1 mM, which was selected on the basis of the maximal solubility of NS in UW. SEK binding was evaluated in ACN due to solubility limitation. The concentrations in the supernatant were again determined with the HPLC-DAD.

### 2.5. Time to Reach Equilibrium

The time to reach the equilibrium state was estimated in batch experiments in UW. A total of 5 mg of each chosen polymer and AC were shaken for 15 min, 30 min, 1 h, 4 h, 8 h and 20 h. The 0.5 mL solutions contained a mixture of SER and the compounds included in Section 2.4 (test mixture), each added at the final concentration of 0.1 mM. The removal percentage was determined by HPLC-DAD.

### 2.6. Binding in WW Matrix: Influence of pH, Salts, and Chemical Oxygen Demand

The behavior of the chosen polymers and AC was observed in WW matrix spiked with the test mixture, again at the final concentration of 0.1 mM. The binding experiments were performed in 3 different matrices: UW, artificial wastewater (WW1) [43], and actual wastewater (WW2) obtained from a Slovenian wastewater treatment plant (WWTP). The WW was filtered (see SM, Section 1.1) before spiking in order to remove particulates and microorganisms that could have influenced the removal. The pH of the WWs was measured using the pH electrode by Wissenschaftlich-Technische Werkstätten GmbH (Weilheim, Germany) and the chemical oxygen demand (COD) was determined on a spectrophotometer using Hach reagents for water analysis, LCK 314 and 514.

We researched the influence of 2 parameters most often reported to influence the binding: pH and the presence of salt ions [44,45,46]. Since the reported pH of WW is between 6 and 8, the performance of the polymers was tested by batch tests in 50 mM phosphate buffer solutions with pH adjusted to 6.0, 7.0, or 8.0 with either a 2 mM HCl or 1 mM NaOH solution. The influence of salt ions was observed by comparing the binding in UW and in NaCl solutions at the concentrations of 0.1 M and 1.0 M.

### 2.7. Upscale Experiment

In order to observe the performance of the materials on a larger scale and at lower concentration of substrate, we packed the material into SPE cartridges by separately weighing 50 mg of MIP, NIP, or AC. MIPs and NIPs were sedimented beforehand in a mixture of MeOH and water (*v/v* = 80/20) four-times for 1.5 h to avoid the loss of material through the frit. For the same reason, AC mesh size 100–400 was used.

The materials were first washed with 5 mL of MeOH and 5 mL of UW water. Then, the cartridges were stacked on top of Oasis HLB cartridges in order to bind the remainder of the unbound compounds. The method used for Oasis HLB conditioning, equilibration, loading, and elution was adapted from our article on photodegradation of SER [9].

A total of 50 mL of WW2 spiked with the mixture of compounds at concentrations of 0.4 µM was loaded at the flow rate of 2 mL min^−1^ on to each material. The solution then flowed directly onto the Oasis HLB cartridge. After loading, the Oasis HLB cartridges were dried for 30 min and then eluted with 3 × 0.6 mL of triethylamine in MeOH. The elution solvent was evaporated, and the extracts were redissolved in 0.5 mL the HPLC mobile phase and filtered through 0.45 µm syringe filters before the HPLC measurements.

### 2.8. Leaching Evaluation

To examine the applicability of developed MIPs as SPE extraction materials, we checked the potential leaching of the template from the MIP. As reported under the upscale experiment (Section 2.7), 50 mg of each MIP was packed in the SPE column, conditioned, loaded, and eluted with 5 mL 1% TFA in MeOH. The extract was dried under nitrogen at 40 °C and the amount of leaching was quantified with a Nexera X2 ultra high performance liquid chromatograph (UHPLC, Schimadzu, Kyoto, Japan) coupled to the hybrid quadrupole-linear ion trap mass spectrometry analyzer QTRAP 4500 (Sciex, Framingham, MA, USA) following the method developed by Gornik et al., (2020a) [9].

### 2.9. Chemical and Morphological Characterization

Fourier transform infrared (FTIR) spectroscopy was performed on IRAffinity-1S (Schimadzu, Kyoto, Japan).

Elemental analysis was performed on a 2400, Series II, CHNS/O Analyzer (Perkin-Elmer, Waltham, MA, USA).

BET surface area analysis was performed with Porozimeter TriStar II (Micromeritics, Norcross, GA, USA).

The morphological characteristics were observed using a scanning electron microscope (SEM). The images were recorded with JSM-7600F (JEOL Ltd., Tokyo, Japan). 

### 2.10. HPLC Measurements

For the determination of SER, NS, SEK, FLU, ESC, PXT, BUP, and BUC in the solutions, we utilized an HPLC-DAD (1260 Infinity Agilent Technologies, Santa Clara, CA, USA). For separation, we applied the column Zorbax Eclipse C-18 column (150 mm × 4.6 mm, 5 µm) (Agilent Technologies, Santa Clara, CA, USA). The injection volume was 10 μL or 20 μL, depending on the tested concentration range. The mobile phases were (A) ACN and (B) 20 mM phosphate buffer at pH 3.70. The gradient started with 70% B for 2 min, decreased to 61% in 13 min, then increased back to 70% B in 0.1 min and was kept as so for 1.5 min. The flow rate was 1 mL·min^−1^. The retention times of the compounds were 3.27 min for BUP, 4.04 min for BUC, 6.20 min for ESC, 8.52 min for PXT, 11.95 min for NS, 12.65 min for FLU, and 13.04 min for SER. SEK was determined with a separate method at flow 2 mL·min^−1^, isocratic elution at 70% A and 30% B. Other parameters coincided with the previous method. SEK eluted at 3.80 min.

## 3. Results and Discussion

All experiments with the exception of the reusability experiments (*n* = 5) were performed in duplicate. The inter-day repeatability reported as the relative standard deviation (RSD) for experiments performed in UW was <5% and in WW < 6%.

### 3.1. MIP Synthesis, Selection, and Reusability

We optimized the polymer composition to tune recognition properties of the material. The initiator (V-65) and cross-linker (EGDMA) were kept constant for all polymerization experiments, while different porogens and co-monomers were added in order to obtain water compatibility, increase capacity, and improve selectivity. The behavior of MIPs compared to their corresponding NIPs was evaluated in batch rebinding experiments performed in water and ACN at different concentrations to generate binding isotherms and calculate the capacity, affinity, and IF. The data we obtained during ACN rebinding experiments enabled us to quantify the binding on the basis only of specific interactions, such as hydrogen bonding, with the minimal non-specific hydrophobic effect [47], while our prime goal was recognition of the investigated compounds in water.

**Figure 2:**
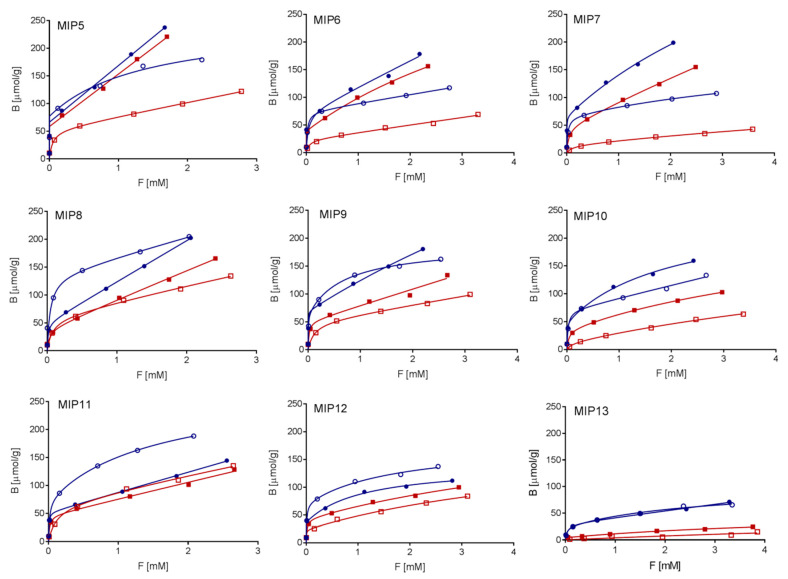
Rebinding isotherms for MIP (blue symbols) and non-imprinted polymer (NIP; red symbols) combinations 5–13 in ultrapure water (UW; full symbols) and acetonitrile (ACN; empty symbols).

**Figure 3:**
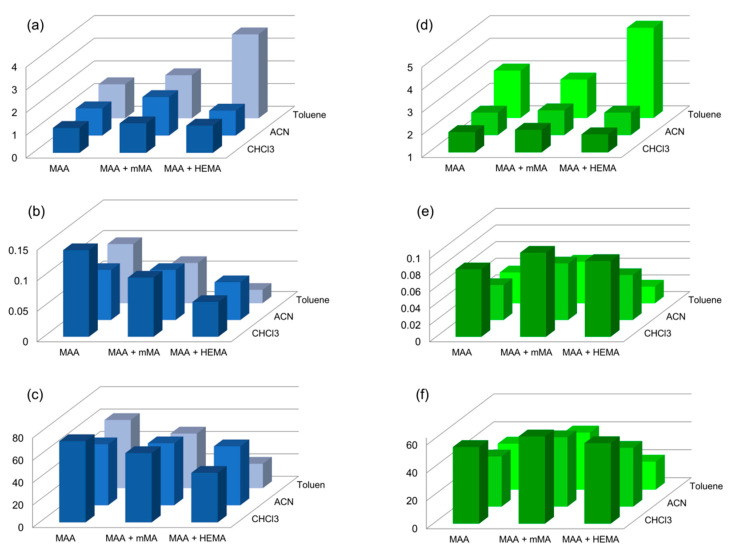
(**a**) The selectivity (imprinting factor, IF), (**b**) the affinity (L·g^−1^), and (**c**) the capacity (mg·g^−1^) of the MIPs in UW in blue. (**d**) The selectivity (IF), (**e**) the affinity (L·g^−1^), and (**f**) the capacity (mg·g^−1^) of the MIPs in ACN in green.

EGDMA in combination with MAA in different porogen solvents is one of the most commonly reported compositions of MIPs to date [30,48,49], including those in MIPs imprinted with SER [50,51]. Unlike in the literature [50,51], we observed no imprinting in MIPs where SER was used in its salt form (MIPs 1–3 in Table 1). Adding the extracted free base form of SER, on the other hand, resulted in successful imprinting. As shown in Figure 2 and Figure 3, we observed higher capacities and affinities in water compared to those in ACN for most tested MIPs, except for MIP11 and MIP12. However, compared to ACN, the Ifs in water were lower in all the cases, indicating loss of selectivity in water. This can be justified by the hydrophobic effect established in polar solvents such as water, and disrupting the formation of hydrogen bonds. In order to improve the recognition abilities in water, we tested the influence of adding co-monomers mMA or HEMA (see examples MIP8–MIP13 in Table 1). Here, the ratios between monomers and cross-linker we applied were based on the results from Dirion et al., (2003). HEMA was chosen on the basis of the reports on improved Ifs in water [47,48], while the mMA was selected as its more non-polar alternative. MIPs with the mMA added into the polymerization mixture (MIP8, MIP9, and MIP10) had a similar IF in ACN, as compared to MIPs 5–7, which were prepared by MAA only (Table 1). However, the Ifs in water were slightly higher for all three materials (Figure 3). The capacities and affinities of MIP8, MIP9, and MIP10 in ACN were higher, yet lower or comparable in water. Compared to MIPs 5–7, adding HEMA as a co-monomer (MIP11, MIP12, and MIP13) did not improve the capacity or affinity of the MIPs in ACN. Additionally, both parameters were noticeably lower in UW. The considerable improvement was, however, observed in IF; the highest was that of MIP13. This high IF is in agreement with the results of Dirion et al., (2013).

As seen in Table 1 and Figure 3, the porogen severely influenced the selectivity, capacity, and affinity of the MIPs. This happens as it affects the stability of the “pre-polymerization complex” (i.e., interactions between functional monomers and the template in the chosen porogen), which plays a crucial role in the imprinting effect. If the porogen disrupts hydrogen bonds between the template and monomers, no specific binding is observed, as can be seen in the case of MeOH (MIP4). On the contrary, using a more non-polar aprotic porogen, the pre-polymerization complex is stabilized, resulting in higher IF, which we showed in MIPs 7, 10, and 13 synthetized in toluene, as compared to those synthetized in ACN (MIPs 6, 9, 12) or CHCl_3_ (MIPs 5, 8, 11) (Table 1, Figure 3a,d) [47].

Determining rebinding characteristics allowed us to select three most promising materials for further testing. In terms of capacity and affinity in water, the material MIP5 was chosen. MIP13 was chosen for its highest IF. Lastly, MIP9 was chosen because it combines satisfactory selectivity, capacity, and affinity in both water and ACN. The chosen polymers were reusable, with the maximum observed decrease in the capacity for SER in four consecutive rebinding experiments being only 2%.

### 3.2. Cross-Reactivity

We determined the cross-reactivity of three selected MIPs for the following antidepressants and structurally related compounds (Figure 1): BUC, BUP, ESC, PXT, NS, SEK, and FLU. While SEK, the metabolite of SER, was also initially included, it however showed very poor binding in ACN and no observed selectivity for any of the three MIPs. Its binding will therefore be based on non-specific interactions only. As for its poor solubility in water, it was thus excluded from further testing.

The results of cross-reactivity tests are selectivity factors (α) reported in Table 2. In general, α for each compound were comparable between the three selected MIPs, with the exception of BUP in MIP13. Here, the factor α 3.29, as compared to 9.60 and 9.76 for MIP5 and MIP9, respectively, indicated more cross-reactivity of MIP13 towards BUP. As reported in Table 2, for NS the selectivity factor was below 1, indicating better binding as compared to SER, which is reasoned by the absence of the methyl group in the chemical structure (Figure 1). Among the SSRI compounds, FLU and PXT had the factors slightly above 1, meaning comparable binding, while the factor for ESC varied between 2.7 and 2.9. The fact that ESC was the only SSRI with a tertiary amine in the structure, together with the favorable α for NS, suggests the impact of steric hindrance of the hydrogen bond-forming amino group on cross-reactivity. The size of the binding site seemed to be of lesser importance, considering that PXT and FLU are larger molecules as compared to SER and NS. BUC and BUP showed higher α in all three MIPs, which is justified by them being less structurally related to the SSRI group. Additionally, their amino groups are also sterically more hindered (tert-butyl group and tertiary amine).

In general, the capacities of the three MIPs for SSRIs followed the same pattern as in SER binding. The highest capacity was observed in MIP5, closely followed by MIP9, and with more than half-lower capacities observed in MIP13 (Figure S1). Furthermore, we compared the binding to the corresponding NIPs. The difference between MIP and NIP was the largest in the case of MIP/NIP13 and the lowest in MIP/NIP5 (Table 2).

### 3.3. Time to Reach the Equilibrium

**Figure 4:**
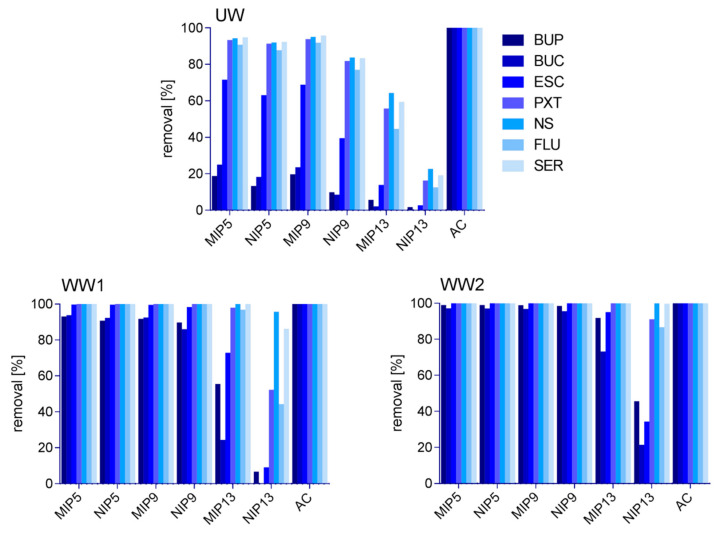
The impact of matrices (UW, artificial wastewater (WW1), and actual wastewater (WW2)) on the performance of MIPs, NIPs, and AC in the batch rebinding test.

The time to reach equilibrium was tested for the chosen polymers and AC. A 0.1 mM test mixture was added. Figure S2 illustrates that for AC, the equilibrium was reached within 1 h; in cases of MIP5 and MIP9, the equilibrium was reached in 4 h; and for MIP13, in 20 h. For the NIPs, similar times to reach the equilibrium were shown as for their corresponding MIPs. As also depicted from Figure 4, AC non-selectively bound all the available compounds until their concentrations in the solvent reached below the limit of quantification (LOQ ≈ 0.001 mM).

### 3.4. Effect of WW Matrix

With the underlying objective to remove pharmaceuticals from WWs, we tested the capacity of MIPs to bind them. This way, we evaluated the ability of MIPs to be applied as sorbents in WW treatment systems. Aiming to get closer to the conditions during WW treatment, we employed the pH adjusted to 6–8 and simulated actual WW matrix composition. By comparing the results between the binding of BUP, BUC, ESC, PXT, NS, FLU and SER in UW, WW1, and WW2, we observed large differences in the removals of the test compounds (Figure 4), whereas AC removed all the tested compounds in any matrix to below LOQ concentrations (0.001 mM). In contrast with our expectations, as shown in Figure 4, the removal efficiencies of MIPs were lowest in UW and highest in the most complex matrix, WW2. In line with the trends shown in the capacity experiments, MIP5 and MIP9 showed best performance, closely followed by NIP5, NIP9, MIP13, and finally NIP13. By investigating the reason for such behavior, we determined the pH and COD of each inspected matrix. The pH values of UW, WW1, and WW2 were approximately 7, 7.2, and 8.2, respectively, whereas we measured COD at <15 mg·L^−1^ for WW1 (LOQ of the test) and 379 mg·L^−1^ for WW2. On the contrary, the literature reports either no change (up to 690 mg·L^−1^ COD) or a slight decrease in adsorption of their chosen templates to their MIPs at high COD values (over 800 mg·L^−1^) [52,53,54,55]. The MIPs in these cases used similar reagents to those in our synthesis, i.e., MAA and EGDMA, albeit in different ratios, and employed DCM or ACN as porogens and 2,2′-azobisisobutyronitrile (AIBN) as the initiator [52,53,54,55]. Hence, we did not expect the higher COD values to be the cause behind the increased removal.

**Figure 5:**
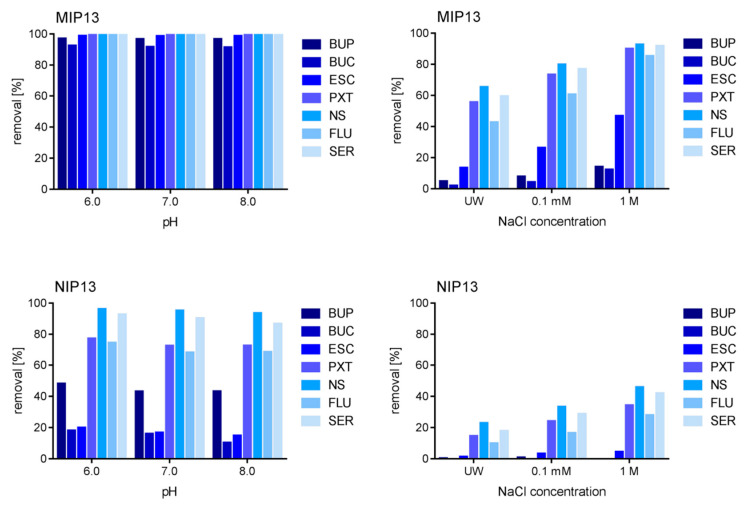
The effect of pH and presence of salt ions in MIP13 and NIP13.

In order to deeper investigate the reasons behind the positive impact of matrix complexity on the removal of pharmaceuticals, we performed the rebinding experiments at different pH values and salt concentrations. Here, the imprinted and non-imprinted polymers showed similar trends, with the most notable differences for MIP and NIP13, as portrayed in Figure 5. The pH in the range of 6 to 8 had almost no influence on the binding with differences below 1%. The only exception was NIP13, with differences between pH 6 and pH 8 ranging up to 7.8%. On the other hand, the increasing salt concentration improved the removal of pharmaceuticals. This finding was further supported by the improved binding found during the pH tests, which were performed in phosphate buffer, as compared to the binding in UW. Our results are consistent with the findings of Kempe and Kempe (2010), where elevated concentrations of salts had a significant influence on the removal of penicillin G from solution and followed the Hofmeister series. As seen in Kempe and Kempe (2010), the higher removal was of non-specific nature, observed in both MIP and NIP [46]. The kosmotropic ions seem to promote the formation of stable interactions between the polymers and tested compounds. Since phosphate ions are more kosmotropic than chloride ions, this would also explain the larger effect in the buffer solutions, despite their lower concentrations [56].

### 3.5. Upscale Experiment

**Figure 6:**
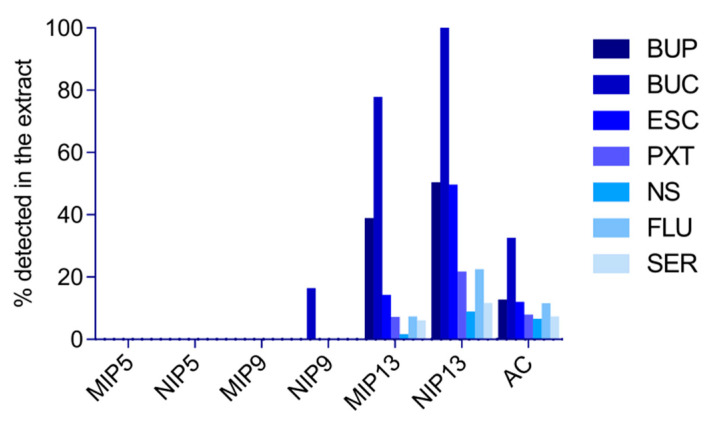
The remainder of compounds detected in the Oasis HLB extracts in the upscale experiment.

The performance of the materials was evaluated as the difference between the initial concentration and the remainder extracted by Oasis HLB SPE. This way, we avoided underestimating the performance of AC, since completely eluting compounds off the AC is a known difficulty [23]. The results on the performance of selected materials in the upscale experiment are shown in Figure 6. The main difference from the batch (mini-MIP) experiments is the less efficient binding to AC (Figure 6). The two main reasons behind this may involve the shorter contact time between the material and WW, or lower capacity of the material due to the non-specific binding of other matrix components. Since in the batch experiment AC showed shortest time to reach equilibrium, the latter is more probable. Furthermore, several reports showed AC performance deteriorating with an increase of matrix complexity (e.g., COD, total dissolved solids) [23,55].

In the Oasis HLB extracts from MIP5, NIP5, and MIP9, none of the investigated pharmaceuticals were detected. On the contrary, as expected, their highest remainder was determined in the Oasis HLB extracts from NIP13, again implying its lowest binding capacity.

While non-specific rebinding is not desired in MIPs that are used, for example, in sample preparation or chromatography, we show here that this phenomenon is favorable in WW treatment. As Le Noir et al., (2007) pointed out, it only becomes a problem if it causes lower capacity and affinity of the selective binding [39]. MIP5 and MIP9 both showed higher capacities compared to MIP13, and even NIP5 and NIP9 performed better under tested conditions. This means that a larger amount of MIP13 would have to be used to achieve the competitive removal efficiencies. However, specific interactions of MIPs will likely play a more important role at higher volumes and more complex matrices. At the same time, we show that the NIPs, which are based on non-specific binding only, are less negatively affected by matrix, as compared to AC, and along with their easy recyclability they could therefore pose a less expensive alternative for the removal of pharmaceuticals.

### 3.6. Leaching

As an alternative to sorption in WW treatment, we also considered the developed MIPs for SPE extraction of environmental samples. As for our hypothesis, MIP could be employed as an SPE sorbent in order to selectively extract targeted compounds, thus reducing the suppressing effect of matrix interferences in further liquid chromatography coupled to mass spectrometry (LC–MS) analysis. Such sorbents may potentially be employed in a highly sensitive analytical method for an ultra-trace level determination of contaminants in WW [57]. MIPs have previously been used for SPE several times [58,59,60,61]. However, given the fact that the template in polymerization (SER) is also the analyte in the LC–MS method, the MIP sorbent would have to pass the “leaching test”, which means that it would have to show a negligible leaching and thus avoid interfering with the assessment of trace-level analytes in the subsequent LC–MS analysis. Leaching of SER from the material was tested on UHPLC-QTRAP, applying the instrumental method developed by Gornik et al., (2020a) [9]. By using 5 mL of 1% TFA in MeOH, we eluted up to 3.5 µg of SER from the MIPs. Alternative methods for template removal, such as microwave or ultrasound-assisted extraction, heating under pressure, or even the use of another acid during Soxhlet extraction, could have lessened the leaching from the MIPs. On the other hand, the more extreme conditions could also have damaged or distorted the imprinted cavities and thus decreased the selectivity, affinity, and capacity of the MIPs [62,63]. Furthermore, the synthesis of MIPs and the subsequent washing procedures triggered the formation of SER transformation products (NS, SEK, hydroxyl-SER) [9], which in turn leached off the materials, thus interfering the environmental analysis. Unfortunately, this makes the material inappropriate for the determination of SER residues including its metabolites and transformation products at trace levels. Finding an appropriate dummy template that would substitute SER and produce a MIP cross-reactive towards SSRI could be a viable solution to such a problem [35]. Nonetheless, the synthetized material can still be applied to SPE of the remaining tested pharmaceuticals (Figure 1).

### 3.7. Characterization

**Figure 7:**
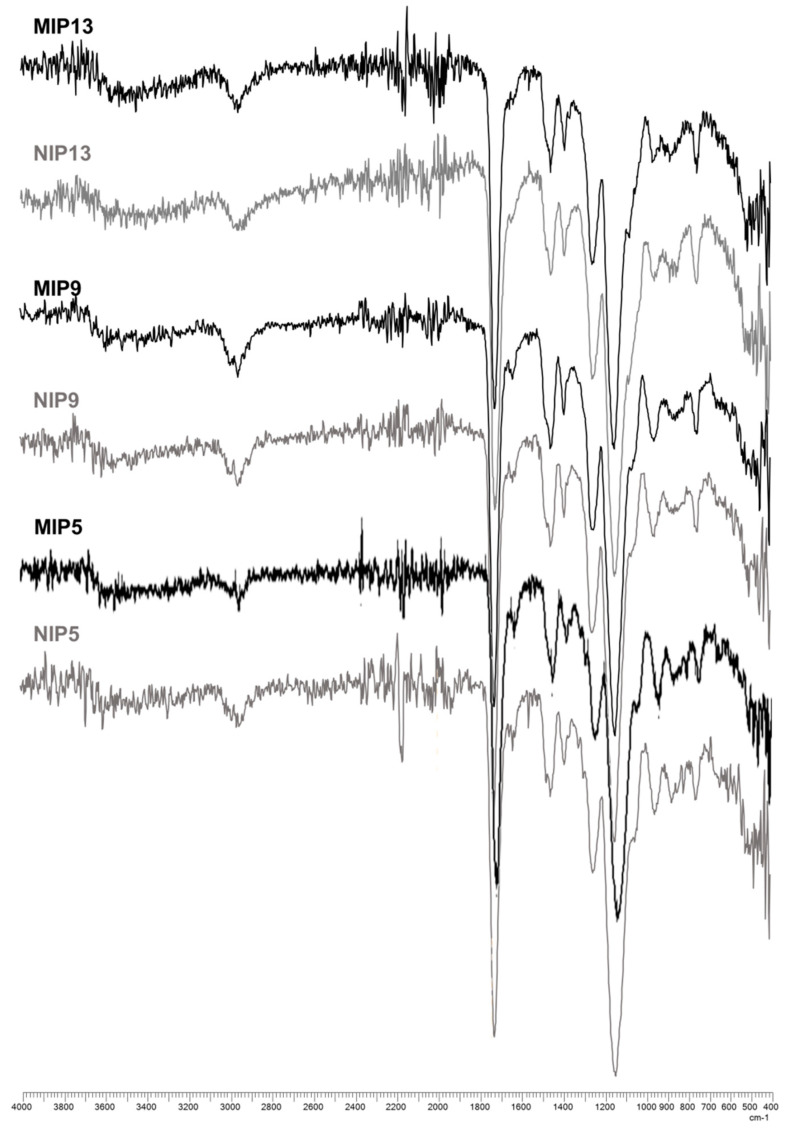
The FTIR spectra of MIPs and NIPs 5, 9, and 13.

The FTIR spectra for the chosen MIP/NIP pairs 5, 9, and 13 can be found in Figure 7. The broad band visible at approximately 3500 cm^−1^ corresponds with the stretching vibration of the hydroxyl group from MAAs COOH group. The stretch bands around 2950 cm^−1^ in all the spectra are part of the C–H vibration present in MAA, mMA, HEMA, and EGDMA. The band around 1720 cm^−1^ represents the vibration from the carboxylic C=O group that can be associated with the C=O groups from MAA, mMA, and EGDMA. The 1250 and 1140 cm^−1^ stretch bands contributed to the stretching of C–O also present in all three compounds. The stretch bands corresponded with the polymerized material. Since the composition of the synthetized materials did not vary strongly, the resulting FTIR spectra were accordingly similar.

The results of the elemental analysis of the MIP and NIP pairs 5, 9, and 13 are reported in Table 3. The results are in accordance with the expected values of the synthetized material. With this measurement, we confirmed that the added reagents reacted in the expected ratio.

The BET surface area, pore size, and pore volume of the MIPs and NIPs are reported in Table 4. As expected, the larger the surface area and pore volume of the tested polymers, the higher the reported capacity and affinity. All three parameters were comparable between MIP and NIP pairs 5 and 9, with BET surface areas for MIP/NIP 5 in the 200 m^2^·g^−1^ range and MIP/NIP 9 at the 100 m^2^·g^−1^ range. However, NIP13 exhibited a more than five times lower BET surface area and pore volume compared to its corresponding MIP (Table 4). A similar difference was observed in MIP and NIP pairs using HEMA as the copolymer in toluene in the research by Dirion et al., (2003). They reported that stronger swelling was observed for the NIPs and similar elution times measured for void markers (acetone or MeOH) in their chromatographic evaluations of the polymers. This indicated a smaller difference between the MIP and NIP in their swollen state.

**Figure 8:**
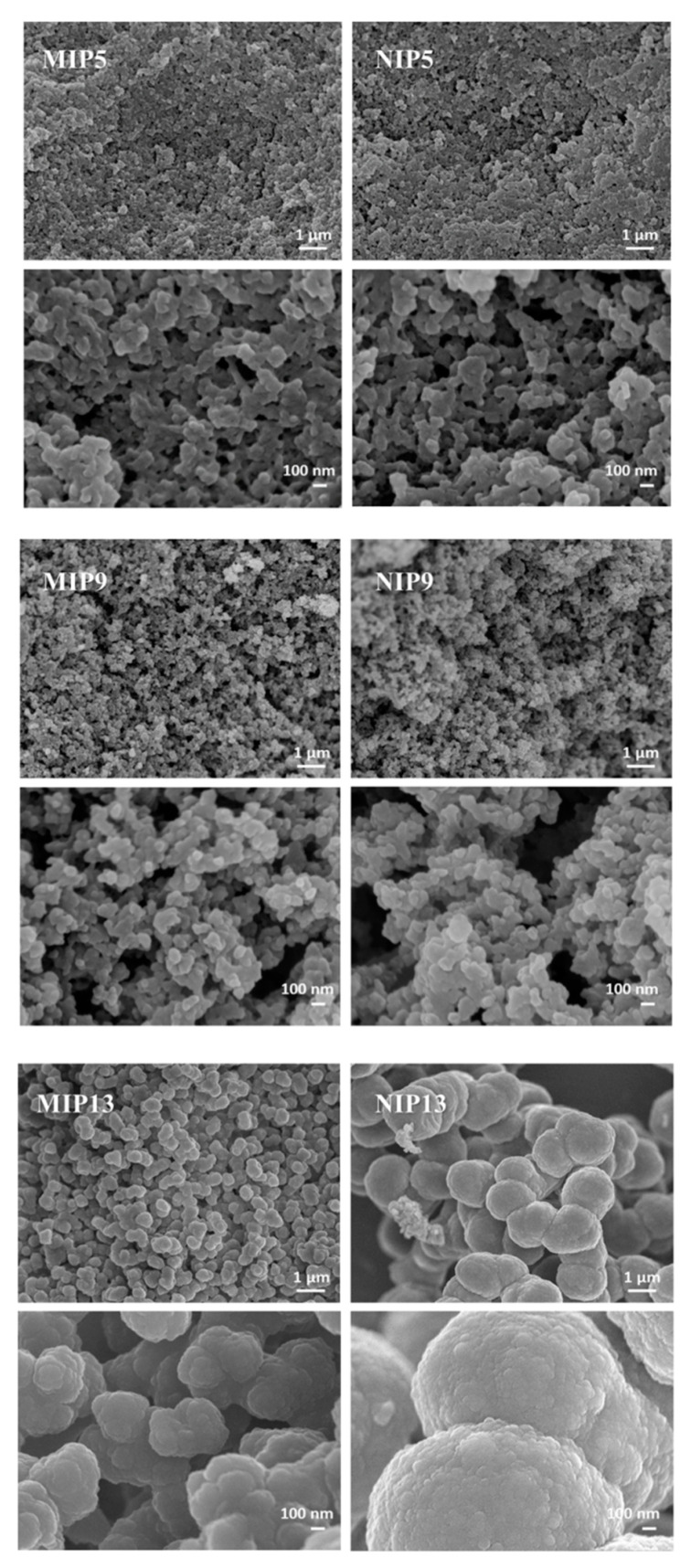
SEM images of MIPs and NIPs 5, 9, and 13.

The SEM images of the surface of our polymers in Figure 8 support the surface area and pore volume measurements. While the morphology of MIP5/NIP5 and MIP9/NIP9 were comparable, the surfaces of MIP13 and NIP13 were dissimilar. These differences in the morphology between MIP and NIP 13 indicate that care should be taken when NIPs are used for the evaluation of MIP selectivity. Comparing a material imprinted with a completely different compound or the determination of α between the template and other compounds can offer more information [35,48].

Compared to AC with a surface area of 1400 m^2^·g^−1^ [64], the surface areas of MIPs and NIPs were 5 to 253 times lower. Nevertheless, some of them showed superior binding characteristics in WW.

## 4. Conclusions

This study investigated the ability of MIPs imprinted with the free base form of SER to remove SSRIs and their metabolites. The functional monomers and porogens revealed a strong impact on the capacity, affinity, and selectivity of the synthetized MIPs. The three selected MIPs showed cross-reactivity towards the SSRIs and the metabolite norsertraline, whereas they bound a lesser amount of the competitors BUP and BUC. Further, the loss of selectivity towards the metabolite SEK was probably due to the loss of the amino group, which was thus found crucial for selective binding to the MIP. The performance of both the imprinted and non-imprinted materials was strongly influenced by the presence of salt ions, which improved their performance in WW. The performance of MIPs was stable throughout WW-relevant pH range 6–8. Compared to AC, the synthetized polymers had at least five times lower surface area and required a longer equilibration time. This slower mass transfer was particularly evident when selective binding was the main driving force behind the removal, as observed in MIP13. However, the capacity in WW for two out of the three tested MIPs surpassed that of AC, and thus both the non-specific and specific interactions showed an important role for the removal from WW. The surface area calculated from the BET isotherm for the MIPs correlated with a higher removal and more non-specific interactions. The advantage of the MIPs is also their reusability that, together with the lower number of regeneration cycles needed due to slower fouling, will cut the costs of the treatment.

Unfortunately, the MIPs were found inappropriate for SPE of samples containing trace levels of SER due to continuous leaching of the template and its degradation products. Future work should include a large-scale experiment confirming the advantages of the synthetized material for the removal of SSRIs from WW.

## Figures and Tables

**Table 1:** Polymer compositions (molar ratio) and ingredients used for the synthesis of molecularly imprinted polymers (MIPs).

Material	Template	MAA	mMA	HEMA	EGDMA	Initiator (V-65)	Porogen
*MIP1*	SER×HCl (1)	4	/	/	20	1 wt % based on total monomers	CHCl_3_
*MIP2*	SER×HCl (1)	4	8	/	12		CHCl_3_
*MIP3*	SER×HCl (1)	4	/	8	12		CHCl_3_
*MIP4*	SER (1)	4	/	/	20		MeOH
*MIP5*	SER (1)	4	/	/	20		CHCl_3_
*MIP6*	SER (1)	4	/	/	20		ACN
*MIP7*	SER (1)	4	/	/	20		toluen
*MIP8*	SER (1)	4	8	/	12		CHCl_3_
*MIP9*	SER (1)	4	8	/	12		ACN
*MIP10*	SER (1)	4	8	/	12		toluen
*MIP11*	SER (1)	4	/	8	12		CHCl_3_
*MIP12*	SER (1)	4	/	8	12		ACN
*MIP13*	SER (1)	4	/	8	12		toluen

**Table 2:** The capacity and selectivity factor of MIP5, MIP9, and MIP13 and the difference in binding of each compound between MIP and the corresponding NIP at 1 mM concentration of each tested analyte.

	MIP5			MIP9			MIP13		
Compound	Capacity (mg·g^−1^)	Selectivity Factor (α)	% (MIP-NIP)	Capacity (mg·g^−1^)	Selectivity Factor (α)	% (MIP-NIP)	Capacity (mg·g^−1^)	Selectivity Factor (α)	% (MIP-NIP)
*SER*	26.6 ± 0.6	1.0	8.4	24.4 ± 0.4	1.0	17.4	11.0 ± 0.3	1.0	25.6
*NS*	26.3 ± 0.3	0.8	7.2	23.1 ± 0.4	1.0	17.1	13.0 ± 0.1	0.7	33.5
*FLU*	25.3 ± 0.6	1.3	4.9	22.4 ± 0.4	1.2	16.2	10.3 ± 0.2	1.1	25.3
*ESC*	20.3 ± 0.5	2.9	4.7	18.8 ± 0.6	2.7	14.8	5.5 ± 0.2	2.8	10.8
*PXT*	27.3 ± 0.1	1.0	3.4	26.3 ± 0.4	1.0	16.8	11.3 ± 0.1	1.1	25.0
*BUP*	7.9 ± 0.1	9.6	2.2	6.5 ± 0.3	9.8	8.0	3.5 ± 0.3	3.3	12.3
*BUC*	10.3 ± 0.3	8.7	9.4	9.7 ± 0.3	7.0	9.4	2.2 ± 0.4	7.0	6.1

**Table 3:** Results of the elemental analysis for MIPs and NIPs 5, 9, and 13.

**MIP 5**	**% C**	**% H**	**NIP 5**	**% C**	**% H**
Theoretical	60.21	7.11	Theoretical	60.21	7.11
Actual	59.56	7.81	Actual	59.68	8
Deviation	0.65	−0.7	Deviation	0.53	−0.89
**MIP 9**	**% C**	**% H**	**NIP 9**	**% C**	**% H**
Theoretical	59.99	7.28	Theoretical	59.99	7.28
Actual	59.55	8.18	Actual	60.2	8.25
Deviation	0.44	−0.9	Deviation	−0.21	−0.97
**MIP 13**	**% C**	**% H**	**NIP 13**	**% C**	**% H**
Theoretical	59.27	7.23	Theoretical	59.27	7.23
Actual	57.00	7.60	Actual	58.03	8.17
Deviation	2.27	−0.37	Deviation	1.24	−0.94

**Table 4:** Brunauer–Emmett–Teller (BET) surface area of MIPs and NIPs 5, 9, and 13.

Material	BET Area (m^2^·g^−1^)	Pore Size (nm)	Pore Volume (cm^3^·g^−1^)
*MIP5*	193.8	7.7	0.374061
*NIP5*	262.1	7.2	0.470623
*MIP9*	136.0	10.3	0.349167
*NIP9*	125.7	9.6	0.300946
*MIP13*	27.4	7.6	0.051835
*NIP13*	5.5	6.6	0.009074

## Supplementary Materials

The following are available online at https://www.mdpi.com/2073-4360/13/1/120/s1: List of standards, chemicals, and materials; Standard solution preparation; Pre-preparation of the ingredients. Figure S1: Cross-reactivity of MIP5, MIP9, and MIP13 in UW. Figure S2: Time to reach the equilibrium for MIP5, MIP9, MIP13, and activated carbon (AC).
