# Supplementary material for: Molecularly Imprinted Polymers for the Removal of Antide-Pressants from Contaminated Wastewater
Source: Polymers (Basel). 2020 Dec 30;13(1):120. doi: 10.3390/polym13010120 (PMC7794900; doi:10.3390/polym13010120)
Supplement: Supplementary file 1 [file polymers-13-00120-s001.pdf]

polymers

MDPI

Molecularly Imprinted Polymers for the Removal of Antidepressants from Contaminated Wastewater

Tjasa Gornik [1,2], Sudhirkumar Shinde [3,4], Lea Lamovšek [5], Maja Koblar [2,6], Ester Heath [1,2], Börje Sellergren [3] and Tina Kosjek [1,2,*]

[1] Department of Environmental Sciences, Jozef Stefan Institute, Jamova 39, 1000 Ljubljana, Slovenia; tjasa.gornik@ijs.si (T.G.); ester.heath@ijs.si (E.H.)
[2] Jozef Stefan International Postgraduate School, Jamova 39, 1000 Ljubljana, Slovenia; maja.koblar@ijs.si (M.K.)
[3] Department of Biomedical Sciences and Biofilms-Research Center for Biointerfaces (BRC), Faculty of Health and Society, Malmö University, 20506 Malmö, Sweden; sudhirshinde1@gmail.com (S.S.); borje.sellergren@mau.se (B.S.)
[4] School of Chemistry and Chemical Engineering, Queens University Belfast, BT9 5AG Belfast, United Kingdom
[5] Department of Biopharmacy and Pharmacokinetics, Faculty of Pharmacy, University of Ljubljana, Askerceva 7, 1000 Ljubljana, Slovenia; lealamovsek@gmail.com
[6] Center for Electron Microscopy and Microanalysis (CEMM), Jamova 39, 1000 Ljubljana, Slovenia
* Correspondence: tina.kosjek@ijs.si; Tel.: +38614773288

1. Materials and methods

1.1. List of Standards, Chemicals, and Materials

Sertraline hydrochloride (SER, (1S,4S)-4-(3,4-dichlorophenyl)-*N*-methyl-1,2,3,4-tetrahydronaphthalen-1-amine, 98%) was purchased from Advanced Technology and Industrial Co., Ltd (Hong Kong, China). Norsertraline hydrochloride (NS, 99.1%) was obtained from LGC (Teddington, UK) and 4-(3,4-dichlorophenyl)-3,4-dihydronaphthalen-1(2H)-one (SEK, 98%) from TCI (Tokyo, China). Fluoxetine hydrochloride (FLU) and escitalopram oxalate (ESC) were donated from the pharmaceutical industry (purity >98%). Anhydrous acetonitrile (ACN, 99.8%), anhydrous toluene (99.8%), aluminium oxide (Al_2O_3), sodium bicarbonate (Na_2CO_3, >99.5%), methacrylic acid (MAA, 99%), acrylic acid (AA, 99%), trifluoroacetic acid (TFA, 99%), ethylene glycol dimethacrylate (EGDMA, 98%), triethylamine (TEA, 99.5% purity), acetic acid (99.8% purity), formic acid (FA, >98%), bupropion hydrochloride (BUP, 99.9%), bupivacaine hydrochloride (BUC, ≤ 100%), methyl methacrylate (mMA), 2-hydroxyethyl methacrylate (HEMA), and activated carbon (AC, mesh size 100-400) were obtained from Sigma-Aldrich (St. Louis, MO, USA). Acros Organics (Geel, Belgium) provided extra dry chloroform ($CHCl_3$, 99.9%). Anhydrous methanol (MeOH, 99.9%) was purchased from Alfa Aesar (Karlsruhe, Germany). 2,2'-Azobis(2,4-dimethyl valeronitrile) (V-65) was provided by FUJIFILM Wako Chemicals (Neuss, Germany). NaH_2PO_4 x H_2O (99–102%) and ortho-phosphoric acid (85%) were supplied by Merck (Darmstadt, Germany). Ethyl acetate (EtAc, 99.9%) and sodium chloride (NaCl) were provided by VWR Chemicals (Radnor, Pennsylvania, USA). The solvents methanol (MeOH, high-performance liquid chromatography (HPLC) reagent), dichloromethane (DCM, ultra resi-analyzed reagent), and acetonitrile (ACN, HPLC reagent) were purchased from J. T. Baker (Deventer, Netherlands). For the preparation of aqueous solutions and for HPLC analysis, we used ultrapure water (UW, 18.2 MΩ.cm). Oasis HLB (60 mg, 3 cc) cartridges were provided by Waters (Milford, MA, USA). Wastewater samples were filtered through GF-2 (Macherey-Nagel, Düren, Germany) and 1.2 μm cellulose nitrate filters (Sartorius Göttingen, Germany).

1.2. Standard Solution Preparation

The standard solutions of SER, NS, FLU, ESC, PXT, BUP, and BUC salts in water and SER free base form solutions in ACN were prepared using HPLC-grade ACN and UW. All the solutions were prepared at 4 mM concentration, with the exception of the NS water solution prepared at 1 mM due to its poor solubility, even in the salt form. The water and ACN standard solutions were stored at 2-8°C for up to 2 days.

1.3. Pre-preparation of the Ingredients

EGDMA, mMA, HEMA, and MAA monomer were treated with basic alumina to remove inhibitors. After basic alumina treatment, the purified monomer was stored at 4°C.

SER is not commercially available in its free base form and therefore had to be transformed by extraction. We added 900 mg of SER×HCl to 200 mL of UW and added the saturated solution of Na_2CO_3 to adjust the pH to11.5. We extracted

SER with 3 x 150 mL of EtAc, which we then washed twice with water and once with brine (saturated solution of NaCl), and then we added Na_2SO_4 to dry the organic solvent. EtAc was removed by evaporation in rotavapor. The product was dried overnight under vacuum and stored in the freezer at - 20°C.

2. Binding Results

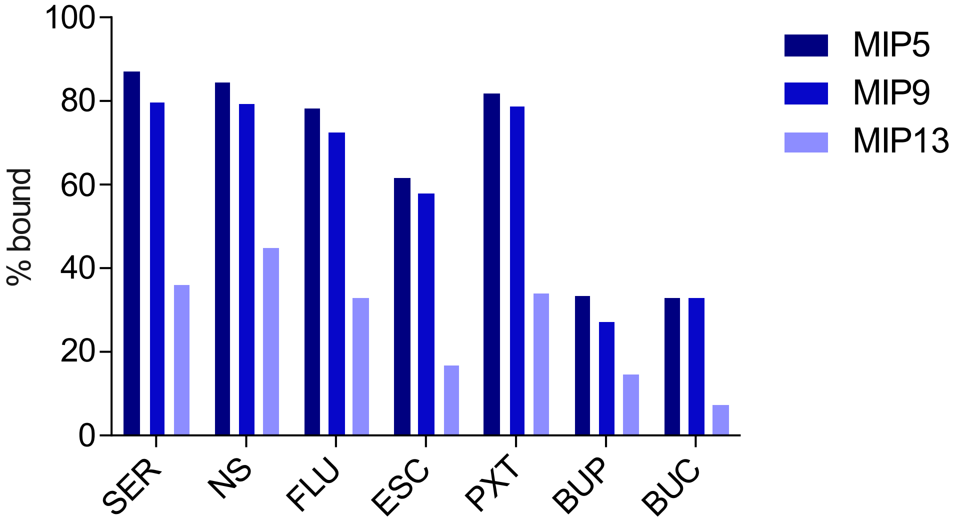

Figure S1. Cross-reactivity of MIP5, MIP9, and MIP13 in UW.

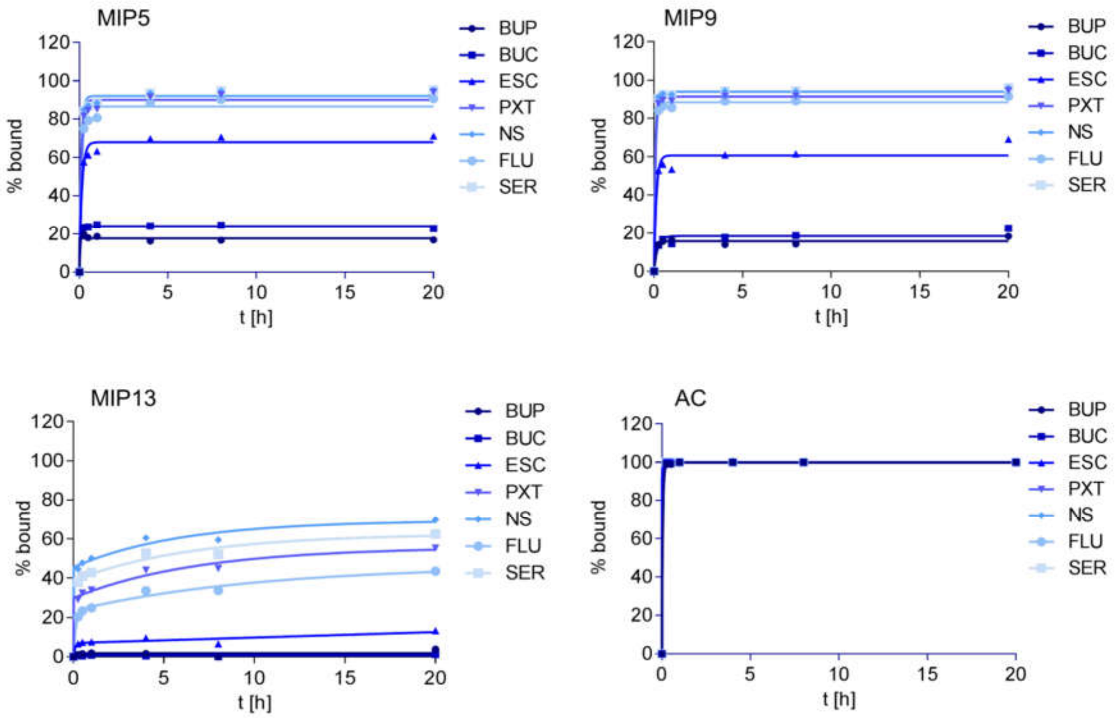

Figure S2. Time to reach the equilibrium for MIP5, MIP9, MIP13, and activated carbon (AC).
